# Quality of life of patients treated with robotic surgery in the oral and maxillofacial region: a scoping review of empirical evidence

**DOI:** 10.1186/s12903-024-04035-w

**Published:** 2024-02-26

**Authors:** Dhanushka Leuke Bandara, Kehinde Kazeem Kanmodi, Afeez Abolarinwa Salami, Timothy Olukunle Aladelusi, Ayodhya Chandrasiri, Jimoh Amzat, Ruwan Duminda Jayasinghe

**Affiliations:** 1 Department of Oral Medicine and Periodontology, University of Peradeniya, Peradeniya, Sri Lanka; 2 School of Dentistry, University of Rwanda, Kigali, Rwanda; 3 Faculty of Dentistry, University of Puthisastra, Phnom Penh, Cambodia; 4 Campaign for Head and Neck Cancer Education (CHANCE) Programme, Cephas Health Research Initiative Inc, Ibadan, Nigeria; 5 School of Health and Life Sciences, Teesside University, Middlesbrough, UK; 6 Department of Oral and Maxillofacial Surgery, University College Hospital, Ibadan, Nigeria; 7 Department of Oral and Maxillofacial Surgery, University of Peradeniya, Peradeniya, Sri Lanka; 8 Department of Sociology, Usmanu Danfodiyo University, Sokoto, Nigeria; 9 Department of Sociology, University of Johannesburg, Johannesburg, South Africa

**Keywords:** Scoping review, Robotic surgery, Outcomes, Quality of life, Dental, Maxillofacial

## Abstract

**Background:**

There is a blooming trend in the application of robotic surgery in oral and maxillofacial care, and different studies had evaluated the quality of life (QoL) outcomes among patients who underwent robotic surgery in the oral and maxillofacial region. However, empirical evidence on the QoL outcomes from these procedures is yet to be mapped. Thus, this study was conducted to evaluate the available scientific evidence and gaps concerning the QoL outcomes of patients treated with robotic surgery in the oral and maxillofacial region.

**Methods:**

This study adopted a scoping review design, and it was conducted and reported based on the Arksey and O’Malley, PRISMA-ScR, and AMSTAR-2 guidelines. SCOPUS, PubMed, CINAHL Complete, and APA PsycINFO were searched to retrieve relevant literature. Using Rayyan software, the retrieved literature were deduplicated, and screened based on the review’s eligibility criteria. Only the eligible articles were included in the review. From the included articles, relevant data were charted, collated, and summarized.

**Results:**

A total of 123 literature were retrieved from the literature search. After deduplication and screening, only 18 heterogeneous original articles were included in the review. A total of 771 transoral robotic surgeries (TORSs) were reported in these articles, and the TORSs were conducted on patients with oropharyngeal carcinomas (OPC), recurrent tonsillitis, and obstructive sleep apnoea (OSA). In total, 20 different QoL instruments were used in these articles to assess patients’ QoL outcomes, and the most used instrument was the MD Anderson Dysphagia Inventory Questionnaire (MDADI). Physical functions related to swallowing, speech and salivary functions were the most assessed QoL aspects. TORS was reported to result in improved QOL in patients with OPC, OSA, and recurrent tonsillitis, most significantly within the first postoperative year. Notably, the site of the lesion, involvement of neck dissections and the characteristics of the adjuvant therapy seemed to affect the QOL outcome in patients with OPC.

**Conclusion:**

Compared to the conventional treatment modalities, TORS has demonstrated better QoL, mostly in the domains related to oral functions such as swallowing and speech, among patients treated with such. This improvement was most evident within the initial post-operative year.

**Supplementary Information:**

The online version contains supplementary material available at 10.1186/s12903-024-04035-w.

## Background

Robotic surgery is a minimally invasive operative technology that has been shown to provide precise navigation through anatomical orifices, preserving vital structures [1, 2]. Thus, this surgical approach has mitigated most of the complications in conventional open surgical approaches, offering limited short-term side effects. The technical spectrum of robotic surgical equipment could range from small wristed instruments attached to a robotic arm to completely autonomous systems which can perform the procedure independently [3]. They also provide three-dimensional vision, improved surgical dexterity, facilities for telesurgery, better ergonomics and enhanced hand–eye coordination [4].

Complex anatomy in the oral and maxillofacial region causes a significant challenge for conventional surgical approaches. Robotic surgery in the oral and maxillofacial region has been used for various applications such as in cancer treatments, thyroid and parathyroid disease, skull base pathologies and in the management of obstructive sleep apnea (OSA) [5]. One of the main complications observed in open procedures is the amount of tissue trauma that could occur due to the large surgical incisions. Moreover, they could result in functional impairment, impaired self-esteem, and decreased quality of life [6]. In contrast, robotic surgery has been shown to cause less post-operative pain and low postoperative infections thus, providing a rapid recovery with better cosmetic outcomes [6].

According to the definition proposed by the World Health Organization, quality of life (QOL) means the “individual's perception of their position in life in the context of the culture and value system in which they live and in relation to their goals, expectations, standards and concerns” [7]. In the health sector, patient-reported QOL outcomes help to facilitate the evaluation of the impact of a disease or treatment procedure, and allow comparison among different treatment strategies [3]. Thus, in an era of minimally invasive concepts, the evaluation of robotic surgery from the perspective of QOL is important. However, QOL outcomes could differ according to the application, anatomical regions, treatment modalities and disease extension.

In relation to robotic surgeries in the oral and maxillofacial region, the available information is unclear regarding the effect of such surgery on QOL outcomes [8–10]. Although several studies that have demonstrated evidence concerning QOL outcomes on different health conditions; no study is known to have mapped the empirical evidence concerning the QOL outcomes of patients who underwent robotic surgery in the oral and maxillofacial region. Hence, there is a need for such review. This scoping review aimed to map the existing scientific evidence, and to identify evidence gaps, concerning the QOL of patients who underwent robotic surgery in the oral and maxillofacial region.

## Methods

### Design

This scoping review was used to identify the gaps and provide an overview of the available evidence on the quality of life aspect of patients who underwent robotic surgeries in the oral and maxillofacial region. The research design developed by Arksey and O’Malley (2005) was used for the review [11]. The review was reported according to the Preferred Reporting Items for Systematic Reviews and Meta-analysis extension for conducting Scoping Reviews (PRISMA-ScR) [12]. Additionally, the guidelines in the AMSTAR 2 checklist were used to ensure the quality of the scoping review methodology and reporting process.

### Identification of the research question

The scoping review’s question was: “What are the available scientific evidence and knowledge gaps in assessing the quality of life of patients treated with robotic surgery in the oral and maxillofacial region?”.

### Identification of relevant literature

To identify all relevant literature, a systematic search was performed across four databases on 21 January 2023: SCOPUS, PubMed, CINAHL Complete (via EBSCOHost interface), and APA PsycINFO (via EBSCOHost interface) to scoop out all literature addressing the review question. The search was conducted, with the aid of the Boolean operators “OR” and “AND” using the following search terms: “robot”, “robotic”, “dental surgery”, “oral surgery”, “maxillofacial surgery”, “periodontal surgery”, “quality of life”, and “wellbeing”. The search field was focused on titles, abstracts, and keywords to identify only relevant literature. Tables S1 to S3 (in the Supplementary file) depict the search strings of the literature search strategy for each of the databases used.

### Selection of literature

All retrieved literature was imported into the Rayyan software for deduplication. After deduplication, the single-entry literature (deduplicated copies) was screened for eligibility for inclusion in the scoping review. Eligibility for inclusion in this review was based on the following criteria:

#### Inclusion criteria

- Literature that was peer-reviewed journal articles.
- Literature that was published in English.
- Literature reporting empirical research findings on quality of life of oral and maxillofacial patients treated with robotic surgery.
- Literature with accessible full texts.

#### Exclusion criteria

- Literature that was published in non-peer-reviewed journals.
- Peer-reviewed journal literature that did not report empirical data e.g. reviews, editorials, commentaries, etc.
- Literature that was not published in English.
- Literature reporting empirical research findings on quality of life of patients treated with nonrobotic surgery in the Oral and maxillofacial region.
- Literature reporting empirical research findings on the quality of life of nonoral and non-maxillofacial patients treated with robotic surgery.
- Literature without accessible full texts (in this context, literature with inaccessible full text was considered non-open access literature whose full text was not received within four weeks of its request from the corresponding author or the British Inter-Library Loan).

The screening process had two stages and was based on the above inclusion and exclusion criteria. Titles and abstracts screening was performed in the first stage to exclude all non-relevant literature. In the second stage, the full texts of all those studies that were not excluded in the first stage were evaluated for relevance. Only those studies that met all the inclusion criteria were considered eligible for inclusion in the review. Importantly, each stage of the screening process was performed by two independent reviewers who were dental surgeons (the first stage by KKK and AAS and the second stage by DLB and AAS). In situations of conflicts in the inclusion/exclusion of a study, they were resolved through critical discussions between the reviewers. The full texts of the included literature were shared with senior experts (who are professors and coauthors in this scoping review: RDJ and JA) for their review. Literature was retained when there was consensus between the experts and the initial reviewers. The final consensus document was shared with all authors for further review. Any disagreement on studies to include or exclude was resolved by the involvement of the entire team. No authors or institutions were contacted to identify additional sources.

### Data charting

From the included literature, data concerning the author names, publication year, country of origin with the robotic system used, study design, study population attributes (disease/application characteristics), QOL instruments, review intervals and the main outcomes were extracted, using a bespoke data extraction sheet (Table 1).

**Table 1:** Summary of the included studies

**No**	**Authors (Year)**	**Country (Robotic system)**	**Study Design**	**Population Characteristics**	**Conditions Treated**	**Assessed QOL domains & QOL instruments**	**Duration of the assessment**	**Outcomes & limitations**
1	Vicini et al. (2010) [13]	Italy [da Vinci Robot (Intuitive Surgical Inc., Sunnyvale, CA, USA)]	Retrospective cohort study	10 patients (8 males [80%] and 2 females [10%]) underwent TOR tongue base reduction, with or without additional procedures (Eg: Septoplasty/inferior turbinate reduction/supraglottoplasty/uvulopalatopharyngoplasty/ethmoidectomy)	Patients with OSA-hypopnoea syndrome primarily due to tongue base hypertrophy (OSAHS)	SF-36 Health Survey	The study duration ranged from 3 to 10 months with a mean follow-up period of 6 months	The post-operative polysomnographic results were favourable, with a mean postoperative Apnoea-Hypopnoea Index of 20.6 (± 17.3 SD). Additionally, the functional outcomes related to pain, swallowing, and quality of life yielded promising results. There were rare occurrences of complications
2	Genden et al. (2011) [14]	USA [da Vinci surgical system (Intuitive Surgical, Sunnyvale, CA, USA)]	Prospective non-randomized case–control study	30 patients (26 males [87%] and 4 females [13%]) with squamous cell CA underwent TORS with or without adjuvant therapy. A comparison group of 26 patients (20 males [77%] and 6 females [23%]) underwent definitive CRT	Base of the tongue CA (11) [37%], Tonsillar CA (11) [37%], Oropharyngeal CA (04) [13%], Soft palate CA (01) [3%], Retromolar trigone CA (01) [3%], Laryngeal CA (01) [3%], Hypopharyngeal CA (01) [3%]	i) Performance Status Scale for Head and Neck Cancer (PSS-HN) ii) Functional Oral Intake Score (FOIS)	Evaluations were done immediately prior to the treatment and within the post-treatment follow-up care, at 2-week and 3-month intervals for 1 year	Two weeks following treatments, surgical patients exhibited notably improved scores for eating, diet and FOIS compared to the patients who underwent CRT However, at 3, 6, 9, and 12 months post-treatment, no significant difference was observed in eating, speech, diet, and FOIS between the two cohorts. In the CRT group, diet and FOIS remained lower than baseline at 12 months after treatment. Thus, TORS could be associated with superior functional outcomes compared to primary CRT
3	Sinclair et al. (2011) [15]	UK [da Vinci surgical system (Intuitive Surgical, Sunnyvale, CA, USA)]	Prospective non-randomized study	42 patients (29 males [69%] and 13 females [31%]) underwent TORS, with or without postoperative adjuvant therapy. 32 patients [76%] underwent postoperative RT and 13 [31%] underwent chemotherapy	Tonsillar CA (29) [69%], Base of the tongue CA (13) [31%]	M D Anderson Dysphagia Inventory (MDADI) questionnaire	Evaluations were done pre-operatively, immediately post-operatively and at a follow-up visit > 3 months from the surgery. The median postoperative follow-up time was 17 months (ranging from 4–40 months)	Immediately following surgery, average MDADI scores in every domain (global, emotional, physical, and functional) showed a decrease compared to the baseline values. Nonetheless, a consistent and gradual improvement was observed in all domains over time. TORS-assisted resection of oropharyngeal squamous cell CA achieves favourable functional and clinical outcomes. However, some limitations exist in time intervals of data collection and lack of exact control group for comparison
4	Leonhardt et al. (2012) [16]	USA (System not mentioned)	Prospective cohort study	38 patients (28 males [73.7%] and 10 females [26.3%]) underwent treatments under three streams; TORS alone, TORS and RT (22) [57.9%] and TORS and CRT (7) [18.4%]	Oropharyngeal squamous cell carcinoma	i) Short Form (SF-8) Health Survey ii) Performance Status Scale for Head and Neck Cancer Patients (PSS-HN)	Assessments were done at baseline; prior to the surgery, and at 6 and 12 months of follow-up period	A transient deterioration of a number of QOL domains was noted by 6 months. However, all domains returned to normal levels by 12 months. TORS alone had minimal and temporary effects on speech. Surgery and radiation had fewer adverse effects on QOL than when surgery and CRT were adopted for treatment. Small sample size may limit the ability to generalize the conclusions
5	Chen et al. (2015) [17]	USA [da Vinci surgical system (Intuitive Surgical, Sunnyvale, CA, USA)]	Retrospective cohort study	31 patients (26 males [84%] and 5 females [16%]) with oropharyngeal CA were treated by trans-oral CO2 laser microsurgery (16) or robotic surgery (15), followed by postoperative RT. Each patient was matched with a patient who had undergone definitive cisplatin-based chemoradiotherapy and was similarly disease-free	Tonsilllar SCC (16) [52], Base of the tongue SCC (15) [48]	University of Washington Quality of Life (UW-QOL) Instrument	Baseline and 1 year	Out of the functional domains of UW-QUL, a statistical difference was observed between the two groups only in the swallowing domain (*p* = 0.01). However, the mean global QOL scores at one year in both cohorts did not show any statistically significant difference (*p* = 0.47). Thus, a similar quality of life could be observed among patients treated by TORS surgery or CRT. Selection bias, varied disease characteristics and management protocol between comparison groups could be possible confounding factors
6	Mercante et al. (2015) [18]	Italy [da Vinci Robot (Intuitive Surgical Inc., Sunnyvale, CA, USA)]	Prospective cohort study	13 patients (6 females [46%]) who underwent TORS with unilateral or bilateral neck dissection without any adjuvant therapy	T1 or T2 base of the tongue CA	i) FEES: Fiberoptic Endoscopic Evaluation of Swallowing ii) Italian MD Anderson Dysphagia Inventory Questionnaire (MDADI) iii) Dysphagia Score (DS) iv) Italian Voice Handicap Index-10 (VHI-10)	Baseline, at 6 months and 12 months postoperatively	Complete recovery of swallowing occurred at 12 months. The speech was unaffected by the surgical intervention while no significant changes were evident in the patient-reported status of swallowing and voice dysfunction, and related QOL after 12-month follow-up. As possible bias; differences in the compared subsets and the disease characteristics could be mentioned
7	Arora et al. (2016) [19]	UK [da Vinci surgical system (Intuitive Surgical, Sunnyvale, CA, USA)]	Prospective case series	14 patients (13 males [92.9%] and 1 female [7.1%]) underwent tongue base reduction using TORS with or without additional wedge epiglottoplasty. Epiglottoplasty was performed in 10 patients [71.4%]	Patients with OSA not complying with conventional treatments such as continuous positive airway pressure or oral appliances	i) Voice satisfaction using Voice Handicap Index 2 (VHI-2) questionnaire ii) Swallowing—MD Anderson Dysphagia Inventory (MDADI) questionnaire iii) Global quality of life—EQ-5D assessment tool system iv) EQ-VAS	Total duration is four years. Patients were assessed at 2 weeks, 3, 6, 12, 18 and 24 months	Worsening of voice function was observed at the first post-operative day and 2 weeks following surgery (*p* < 0.05). However, the mean score gained the baseline levels by 3 months. The same patterns were observed in swallowing function. The overall QOL was improved in all patients from 3 months onwards. Therefore, TORS of the tongue base with or without epiglottoplasty seems a considerable treatment option for selected patients with OSA. As limitations, small sample size, lack of control group, selection bias and performance bias have been stated
8	Ling et al. (2016) [20]	USA (System not mentioned)	Retrospective observational comparison cohort study	138 patients (115 males and 23 females) who underwent primary TORS with or without adjuvant (chemo)radiotherapy (92) or definitive CRT (46)	Tongue base CA (67), Tonsillar CA (58), Pharyngeal wall CA (4), Soft palate CA (2), Unknown primary (7)	University of Washington Quality of Life—Version 4 (UW-QOL V4) questionnaire	The assessments were done at 1, 6, 12, and 24 months from the completion of TORS or definitive CRT	Patients who underwent definitive TORS experienced notably improved long-term outcomes in the saliva domain. Among patients who received adjuvant therapy, QOL declined in both the saliva and taste domains. Adjuvant therapy was also linked to poorer scores in terms of appearance and recreation at 6 months, speech at 12 and 24 months, and chewing and swallowing at 24 months. Compared to surgery combined with adjuvant therapy, definitive CRT resulted in poorer QOL related to saliva at 1 month and reduced QOL related to chewing at 12 months. However, it is worth noting the significant difference in disease staging between the two groups. Also small sample size and selection bias could have contributed to the outcome
9	Ozbay et al. (2017) [21]	USA [da Vinci surgical system (Intuitive Surgical, Sunnyvale, CA, USA)]	Prospective cohort study	29 patients (27 males [93.1%] and 2 females [6.8%]) underwent TORS and unilateral neck dissection with postoperative RT (13) [44.8%] or postoperative CRT (16) [55.1%]. Among them, 27 (96.4%) were HPV positive	Tonsillar CA (17) [58.6%], Tongue base CA (7) [24.1%], Occult primary (5) [17.2%]	Head and Neck Cancer Inventory (HNCI)	Evaluations were done at baseline preoperatively and at 3 weeks, 3 months, 6 months, and 12 months postoperatively	Compared to the initial baseline values, at three weeks, QOL declined significantly in the eating and speech domains. At three months, these reductions were more significant in all five domains. At six months, improvements were observed only in the speech and aesthetics domains, while other domains and the overall QOL continued to demonstrate reductions. However, at 12 months, all domains showcased enhancements, except for speech and aesthetics domains which showed a return to the baseline level. As possible bias factors; small sample size, lack of long-term follow up data and the absence of non-surgical comparison arm could be highlighted
10	Achim et al. (2018) [22]	USA [da Vinci surgical system (Intuitive Surgical, Sunnyvale, CA, USA)]	Prospective longitudinal cohort study	74 patients (68 males [92%]) underwent partial pharyngectomy or hemiglossectomy via TORS with concomitant neck dissection. Adjuvant CRT was administered for tumours having positive margins and extra-nodal extension while adjuvant RT was given for tumours with perineural invasion, lymphovascular invasion, or pN2a or greater disease. Therefore, the comparisons were done between the groups of patients undergoing TORS only and TORS and adjuvant radiotherapy (TORS + RT) or TORS and chemoradiotherapy (TORS + CRT)	Tonsillar CA (42) [57%], Base of the tongue CA (31) [42%] and carcinoma of unknown primary (1) [1%]	i) Eating Assessment Tool (EAT-10) ii) University of Michigan Head and Neck Quality of Life (HNQOL) Instrument	Collected at baseline, postoperatively between 7–21 days, at 6–12 months intervals and long-term follow-up (> 12 months). The median long-term follow-up was 21 months. For short-term follow-up, the records were taken from 65 patients (88%) while for the long-term follow up only 64 patients (86%) were included	Compared with patients who underwent adjuvant therapy, patients who underwent surgery alone experienced better functional outcomes, especially related to swallowing and speech, with QOL measurements returning to near baseline. However, the study also highlights several limitations such as broad time points for follow up, possible variations in nature and extent of the primary disease, in providing adjuvant therapy and possibility to attend for follow up care due to the coverage of wide geographic area
11	Gallitto et al. (2019) [23]	USA (System not mentioned)	Retrospective cohort study	46 patients (44 males [95.7%] and 2 females [4.4%]) underwent TORS (including unilateral or bilateral neck dissection) with unilateral neck RT (9) or bilateral neck RT (37). 17% of the patients received adjuvant CRT Two major cohorts were compared in this study: i) Patients who were treated with trimodality therapy (surgery + chemotherapy + RT) with bilateral neck radiation, Patients treated with trimodality therapy with ipsilateral neck radiation, sparing the contralateral side	Base of the tongue CA (19) [42.4%], Tonsillar CA (26) [57.8%]	University of Washington Quality of Life (UW-QOL) Questionnaire	Within the first 6 months and after 1 year following completion of chemoradiation therapy	There was no statistically significant difference in overall survival between the two groups. However, unilateral neck radiation was associated with better patient-reported outcomes in salivary function, mood, and anxiety HPV + node-positive T1-T2 non-well-lateralized tonsil or tongue base cancers represent ideal candidates for primary robotic surgery with bilateral neck dissection to identify those who need ipsilateral radiation, by sparing the contralateral neck, since unilateral neck radiation with concurrent chemotherapy provides superior QOL and comparable survival to those undergoing bilateral neck radiation with concurrent chemotherapy. As a major limitation, lack of balance between patients receiving unilateral and bilateral neck RT could be highlighted
12	Lazarus et al. (2019) [24]	USA (System not mentioned)	Prospective cohort study	10 patients (5 males and 5 females) underwent TORS with (9) or without (1) neck dissection. Five patients underwent adjuvant RT. Chemotherapy was not administered to any of the patients	Base of the tongue CA (5), Tonsillar CA (5)	i) MD Anderson Dysphagia Inventory (MDADI) ii) Performance Status Scale (PSS)	Baseline and at 1 month post-operatively	All the patients showed a normal level of swallowing function, ability to eat in public, ability to take a normal diet, normal range of tongue motion and optimal understandability of speech by 1-month post-surgically. However, the small sample size may have led to errors such as selection bias, sampling errors and insufficient power
13	Di Luca et al. (2020) [25]	Italy [da Vinci surgical system (Intuitive Surgical, Sunnyvale, CA, USA)]	Retrospective cohort study	84 patients (16 females [19%] and 68 males [81%]) with recurrent lingual tonsillitis were treated with a lingual tonsil resection using trans-oral robotic surgery. Among them, a cohort of 60 (71.4%) patients was subjected to an assessment of their quality of life after surgery and post-operative dysphagia	Recurrent lingual tonsillitis	i) Glasgow Benefit Inventory (GBI) ii) MD Anderson Dysphagia Inventory (MDADI) questionnaire	The mean clinical follow-up time ranged from 49.6 ± 27.1 months (range 6–109 months)	In GBI, the domains of general, Social and Physical showed mean values of + 49.5 ± 21.5, + 28.8 ± 20.3, and + 65.8 ± 32.1 respectively The average composite MDADI score showed an optimal level of swallowing TORS provides a promising treatment option for recurrent lingual tonsillitis providing improved QoL and swallowing function
14	Xu et al. (2020) [26]	USA (System not mentioned)	Retrospective cohort study	76 patients (62 males [81.6%] and 14 females [18.4%]) with Human papillomavirus–associated oropharynx squamous cell carcinoma who underwent surgery alone (17), surgery with adjuvant (chemo)radiation [S-a (C)XRT] (23), and definitive (chemo)radiation [d (C)XRT] (36)	Tonsillar CA (44) [57.9%], base of the tongue CA (27) [35.5%], Pharyngeal wall CA (1) [1.3%], Soft palate CA (1) [1.3%], Unknown primary (3) [4%] All were HPV-associated stage 1 OPSCC	i) University of Washington Quality of Life (UW-QOL) version 4 ii) European Organization for Research and Treatment of Cancer Quality of Life Questionnaire-Core Module (EORTC QLQ-C30) version 3.0 and its head and neck-specific module (EORTC QLQ-HN35) iii) University of Michigan Xerostomia Questionnaire (XQ) iv) Neck Dissection Impairment Index (NDII)	Median follow-up time of 2.2 years. The participants responded to the questionnaire at different time intervals	Most of the patients perceived their post-treatment overall health-related QOL as good or better, showing statistical equivalence across treatment groups. Better salivary/taste/oral functions and less pain, oral/dental, sexual and financial problems were reported in patients who underwent surgery alone compared to d(C)XRT and S-a(C)XRT patients. S-a(C)XRT patients encountered more problems associated with appearance and cough, compared to d(C)XRT patients. No statistically significant differences in neck or shoulder functions were found between surgical and non-surgical patients However, post-treatment QOL for early-stage HPV + oropharyngeal squamous cell carcinoma patients is usually high regardless of the treatment modality. The differences between S-a(C)XRT and d(C)XRT cohorts are subtle/may be due to other factors. Moreover, it is important to note the limitations in a retrospective study
15	Lee et al. (2022) [27]	USA (System not mentioned)	Retrospective cross-sectional study	37 patients (35 males [94.6%] and 2 females [5.4%]) with HPV + , Oropharyngeal squamous cell CA who underwent neoadjuvant chemotherapy followed by TORS at least 2 years before the study	Tonsillar CA (16) [43.2%], Base of the tongue CA (20) [54.1%], Soft palate CA (1) [2.7%] All cases were HPV +	i) MD Anderson Dysphagia Inventory Questionnaire (MDADI) ii) Functional Oral Intake Score (FOIS)	Median of 3.8 years post-treatment (interquartile range, 2.0–8.6 years)	78.4% (*n* = 29) of patients who were treated with neoadjuvant chemotherapy and TORS achieved optimal long-term swallowing function (MDADI > 80) with a near-normal median MDADI composite score of 98.9. A majority of the patients tolerated total oral intake without restrictions Patients with oropharyngeal squamous cell carcinoma treated with neoadjuvant chemotherapy and TORS may achieve favourable long-term swallowing outcomes, by preventing post-operative radiotherapy. Nevertheless, the pre-treatment MDADI scores were not available to detect the precision changes in the swallowing function
16	Price et al. (2022) [28]	USA (System not mentioned)	Prospective cohort study	79 patients (71 males [89.9%] and 8 females) with Oropharyngeal CA. All underwent surgery (94.9% TORS) with adjuvant CRT under two different regimes. [Cohort A—30 Gy in 1.5-Gy fractions twice a day over 2 weeks with weekly docetaxel (15 mg/m2) if they had intermediate pathological risk factors, Cohort B—36 Gy in 1.8-Gy fractions twice a day over 2 weeks with the same chemotherapy regime if they had extra-nodal extension]	Tongue base CA [41.8%], CA involving tongue base and other areas [5.1%], Tonsillar CA [39.2%], CA involving tonsil and other areas [1.3%], CA involving tonsil and soft palate [1.3%], Tonsil and tongue base CA [11.4%] All were HPV + cases	i) European Quality of Life (Eq-5D) ii) Functional Assessment of Cancer Therapy − Head and Neck Version 4 (FACT-H&N) iii) European Organisation for Research and Treatment of Cancer Head and Neck 35 (EORTC H&N 35) iv) The University of Michigan Xerostomia-related QOL Scale (XeQOLS)	Baseline measurements were taken post-surgically prior to initiating adjuvant radiotherapy (RT) and at 1, 3, 12 and 24 months post-RT	Compared with the baseline levels, both cohorts did not show any significant difference in all the QOL measurements at 12 months. All the assessment tools showed an improvement in QOL and most patients returned to baseline level of function and QOL. Excellent swallow outcomes were maintained with the preservation of global and xerostomia-related QOL. Nevertheless, the study also discusses the possible impact of lack of pre-surgical data on swallowing function and QoL on the overall interpretation
17	Salmon et al. (2022) [29]	USA (System not mentioned)	Prospective cohort study	09 participants (07 males and 02 females) underwent tongue base hemi-resection with or without adjuvant therapy. 04 participants underwent TOR alone while 04 participants underwent adjuvant RT. 02 patients underwent adjuvant chemotherapy, out of which one patient underwent both chemotherapy and RT	Tongue base CA	i) Eating Assessment Tool (EAT-10) ii) Functional Oral Intake Score (FOIS)	At the Baseline and post-operatively at one-week, four-week and 12-week intervals	The scores worsened by 1 week post-operatively but improved within the 4-week and 12-week intervals. Similarly, FOIS had a significant difference with the week 1 evaluation and there were no significant differences with week 4 and 12. Therefore, TORS could make changes in the swallowing-related QOL, and oral intake in the immediate postoperative phase. As limitations of the study followings have been stated: Small sample size, the effect of other confounding factors on functional outcomes, absence of constant follow up for patients with newly diagnosed base of the tongue CA during the preoperative time frame, loss of follow up cases
18	Scott et al. (2022) [30]	Denmark (System not mentioned)	Prospective longitudinal cohort study	44 patients (33 males [75%] and 11 females [25%]), aged 18 years or older, with the following characteristics were included; World Health Organization (WHO) performance stage of 0–2, no evidence of distant metastasis, no previous history of head and neck cancer or RT in the head and neck region 31 patients underwent TORS alone while 13 patients underwent only RT	OPC involving the palatine tonsils (30) [68%], the base of the tongue (11) [25%] and the soft palate (3) [7%]	i) European Organization for Research and Treatment of Cancer Quality of Life Questionnaire Core (EORTC QLQ-C30) ii) European Organization for Research and Treatment of Cancer Quality of Life Questionnaire Core-Head & Neck Module (EORTC QLQ-H&N35) iii) MD Anderson Dysphagia Inventory questionnaire (MDADI)	At baseline, 1 year follow up and 3 years follow up	Although both cohorts showed improvement in swallowing function after one year, the TORS group showed a higher mean composite MDADI score at 3 years compared to the baseline level In the EORTC-C30, the only statistically significant difference between 1- and 3-year scores was an improvement in the physical subscale of 8.6 (*p* = 0.037) for patients treated with RT. It is important to note the differences in the disease characteristics and the management protocols for the comparison groups in this study

*CA* Carcinoma, *OPC* Oro-Pharyngeal Carcinoma, *HPV* Human Papilloma Virus, *RT* Radiotherapy, *CRT* Chemo radiotherapy, *TORS* Tran-Oral Robotic Surgery

### Collation, summary and reporting of results

The extracted data were collated, summarized, and presented in the forms of texts, tables and figures.

## Results

**Fig. 1:**
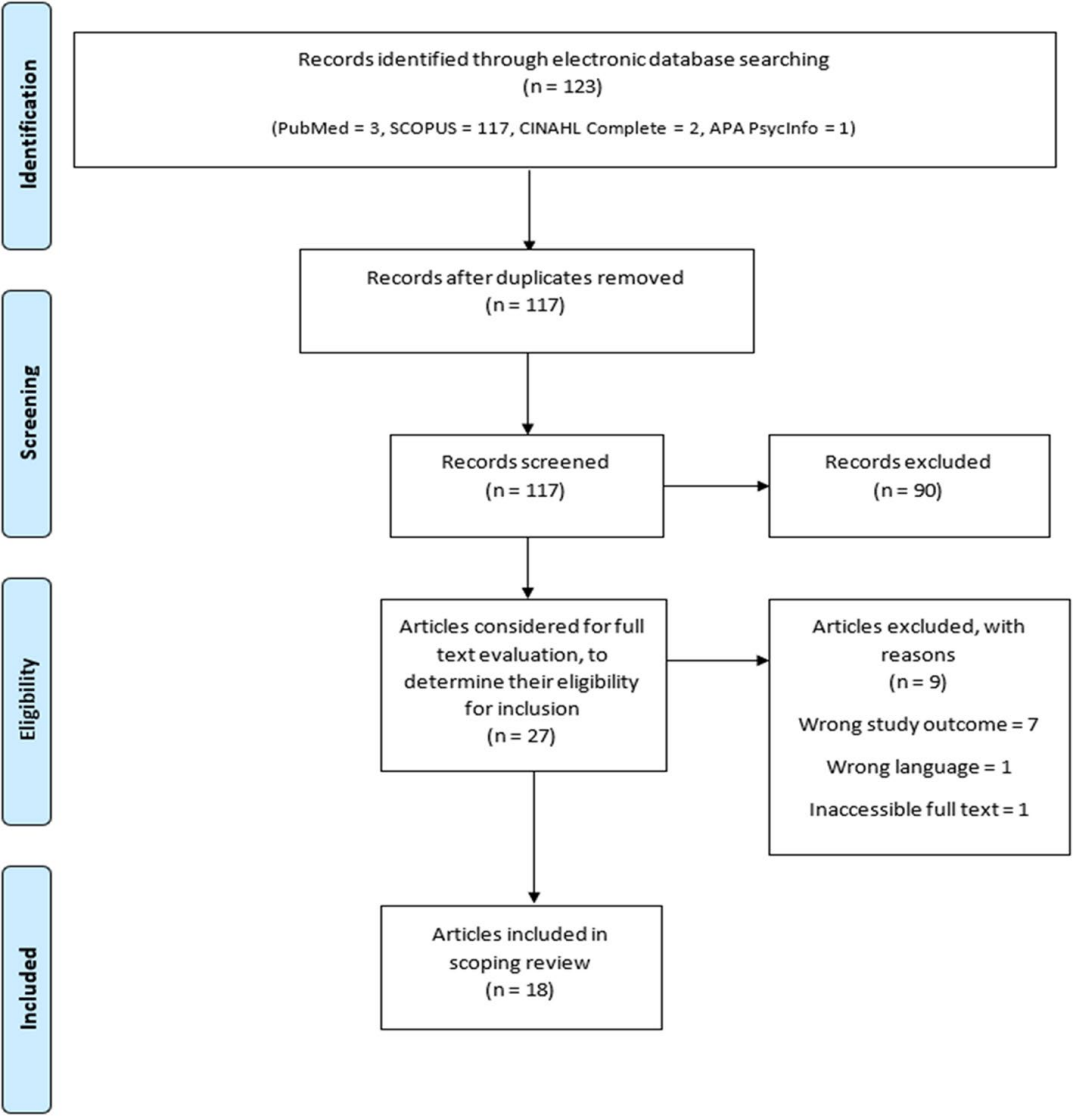
PRISMA flow chart diagram

One hundred and twenty-three papers were retrieved from the database search (PubMed = 3, SCOPUS = 117, CINAHL Complete = 2, APA PsycInfo = 1). Of these 123 papers, 6 were found to be duplicates and were removed. The remaining 117 papers were screened for inclusion in this review. After two-staged screening, only 18 papers (original research articles) were found eligible for inclusion and were included in this review (Fig. 1; Table S4 (Supplementary file)).

### Characteristics of included studies

#### Geographic distribution and study population

**Fig. 2:**
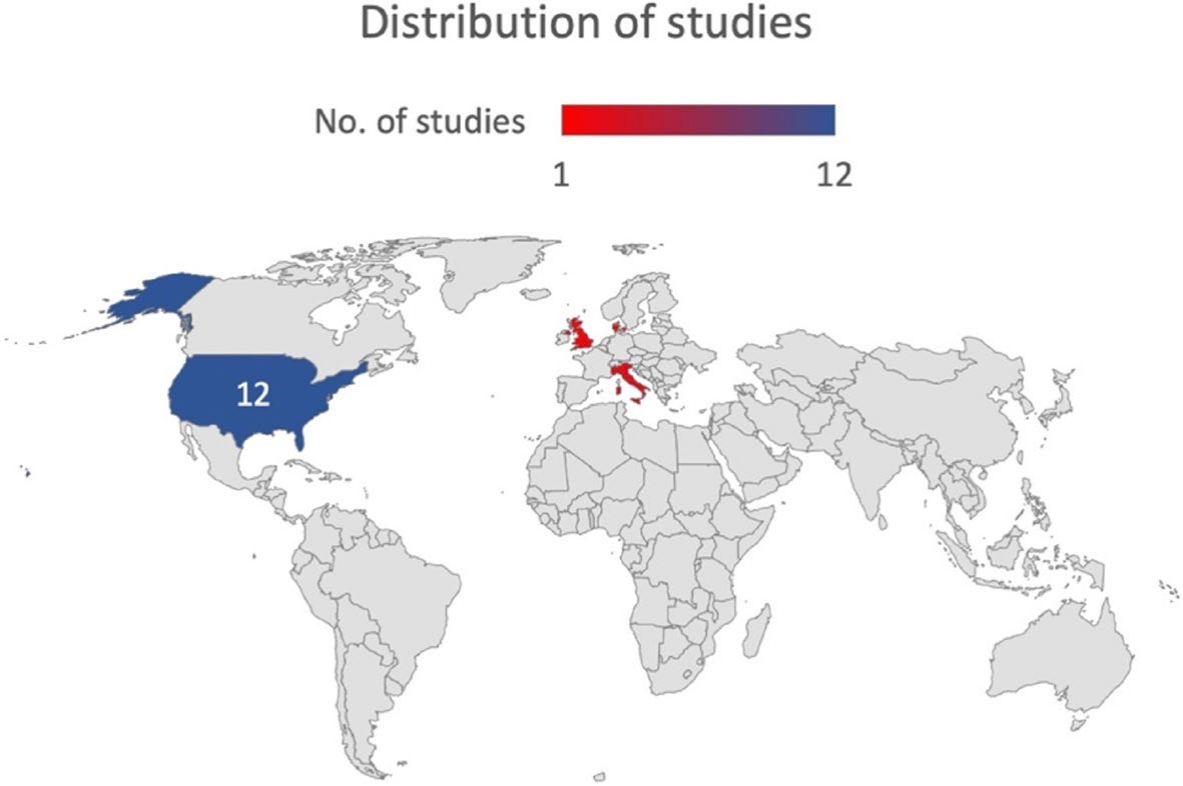
Distribution of the studies depending on the country of origin

In terms of country of origin, all studies were from the USA and Europe and the majority were from the USA (*n* = 12, 66.67%). Others included three from Italy [13, 18, 25], two from the United Kingdom [15, 19] and one from Denmark [30] (Fig. 2). A total of 804 patients were studied in all the reviewed articles with a minimal sample size of 9 participants [29] to a maximum of 138 [20]. The populations mainly varied according to the main pathology and the age groups while in the cohorts of OPC, a significant variation was observed depending on the site of the lesion, human papillomavirus positivity, nodal involvement, and the extent of the lesion.

#### Study designs

According to the study design, 10 articles were prospective study designs (55.6%) [14–16, 18, 21, 22, 24, 28–30] while seven were retrospective studies [13, 17, 20, 23, 25–27]. One study was based on a prospective case series [19]. The follow-up periods varied from a one-month postoperative period to a median of 3.8 years.

### Application of the TORS

In the reviewed articles, a total of 771 TORS procedures were conducted, and they were mainly on the management of OPC (*n* = 15), recurrent tonsillitis (*n* = 1) [25] and OSA (*n* = 2) [13, 19]. Except in nine studies [16, 20, 23, 24, 26–30] in which the robotic system used was not defined, all other studies were based on TORS performed using the da Vinci Robot (Intuitive Surgical Inc., Sunnyvale, CA, USA) system.

### QOL measures

#### Instruments

In total, 20 different instruments were used to assess various aspects of the QOL. Furthermore, some studies used multiple instruments measuring numerous domains. Except in six studies ([13, 15, 17, 20, 21, 23], others used two or more instruments to evaluate the factors that could be related to QOL (Table 2). The most commonly used instrument was the MD Anderson Dysphagia Inventory Questionnaire (MDADI), which evaluates the impact of dysphagia on QOL.

**Table 2:** Different instruments used for the measurements of QOL aspects

	Instrument	Reference/s
1	European Organization for Research and Treatment of Cancer Quality of Life Questionnaire Core (EORTC QLQ-C30)	[30]
		[26]
2	European Organization for Research and Treatment of Cancer Quality of Life Questionnaire Core-Head & Neck Module (EORTC QLQ-H&N35)	[30]
		[28]
		[26]
3	MD Anderson Dysphagia Inventory questionnaire (MDADI)-Swallowing related QoL	[30]
		[27]
		[25]
		[24]
		[19]
		[18]
		[15]
4	University of Washington Quality of Life (UW-QOL) Instrument	[17]
		[26]
		[23]
		[20]
5	Eating Assessment Tool (EAT-10)	[22]
		[29]
6	University of Michigan Head and Neck Quality of Life (HNQOL) Instrument	[22]
7	Functional Oral Intake Score (FOIS)	[29]
		[27]
		[14]
8	European Quality of Life (Eq-5D)	[28]
		[19]
9	Functional Assessment of Cancer Therapy − Head and Neck Version 4 (FACT-H&N)	[28]
10	University of Michigan Xerostomia-related QOL Scale (XeQOLS)	[28]
		[26]
11	Glasgow Benefit Inventory (GBI)	[25]
12	Neck Dissection Impairment Index (NDII)	[26]
13	Performance Status Scale (PSS)	[24]
		[14]
		[16]
14	Head and Neck Cancer Inventory (HNCI)	[21]
15	Voice satisfaction using Voice Handicap Index 2 (VHI-2) questionnaire	[19]
		[18]
16	EQ-VAS	[19]
17	FEES: Fiberoptic Endoscopic Evaluation of Swallowing	[18]
18	Dysphagia Score (DS)	[18]
19	Short Form (SF-8) Health Survey	[16]
20	Short Form Health Survey (SF-36)	[13]

#### QOL domains assessed, outcomes and factors affecting QOL

Of the physical, psychological, and social functions assessed in the included articles, physical functions related to swallowing, speech and salivary functions were the most assessed aspects (Table 2). Moreover, in patients with OPC, compared to other common treatment modalities such as chemoradiotherapy, most of the studies reported better outcomes in swallowing functions in patients who underwent TORS [14, 15, 17, 27, 29, 30] (Table 1). It was also noted that speech is minimally affected in TORS procedures [16, 21] (Table 1). Some studies also noted improvement in overall oral functions including taste, speech, saliva functions and eating [20, 26]. However, a significant difference in QOL outcomes was not observed after one year (Table 1). The location of the lesion, the need for unilateral/bilateral neck dissection and adjunctive therapies such as chemoradiation were the main factors affecting QOL (Table 1).

Among those patients who underwent tonsillectomy and tongue base reduction due to hypertrophic tongue, they also demonstrated better functional outcomes and improved QOL with TORS [13, 19, 25] (Table 1).

## Discussion

This scoping review aimed to evaluate the available scientific evidence and gaps in the quality of life of patients treated with TORS in the oral and maxillofacial region. Within the studied literature, the application of TORS in the oral and maxillofacial region was limited to the management of OPC, OSA and recurrent tonsillitis. The applied robotic system, da Vinci Robot (Intuitive Surgical Inc., Sunnyvale, CA, USA), provides a magnified three-dimensional high-definition view, a more precise incision due to the wristed instruments that could be bent and rotated better than the human hand and a more reproducible approach compared with traditional open surgical techniques [31]. Nevertheless, affordability for the high implementation cost and the availability of training facilities may have contributed to the localization of the TORS to a few countries [32].

Assessment of QOL following a surgery provides a metric-based evaluation of the procedure which could also assist a patient in decision-making [33]. In surgeries involving OPC, QOL assessments are mostly based on the preservation of key functions such as speaking, swallowing, and aesthetics. Most of the studies used validated questionnaires specific for the assessment of post-surgical QOL, such as the EuroQol Health Survey (EQ-5D), European Organization for Research and Treatment of Cancer Quality of Life Questionnaire Core (EORTC QLQ-C30) and SF-36 Health Survey. Similarly, OSA could affect QOL significantly due to snoring, daytime sleepiness and unrestful sleep. Therefore, QOL could be a reliable parameter to evaluate the functional outcomes of a management protocol. Dysphagia, a well-known drawback of surgeries in the head and neck region, has been extensively assessed in this review using various instruments, including the MDADI, EORTC QLQ-H&N35, UW-QOL, and DS.

Although some studies demonstrated no significant difference between the cohorts [24, 26, 28], most of the evaluated studies demonstrated a shorter recovery time to reach baseline or better QOL in patients who underwent TORS. The deterioration in QOL in the presence of adjuvant therapy is also worth noting in decision-making on the suitable treatment option. However, as major gaps in the available literature, the absence of long-term effects of TORS on QOL and the lack of properly designed randomized controlled trials could be highlighted.

The comprehensive search strategy in four large databases and the minimally biased review protocol adhered to in this review were the main strengths. Additionally, the main findings of this review enhanced the literature by emphasizing the outcomes specific to robotic surgeries in the head and neck area and the gaps that need to be addressed in future studies.

Nevertheless, the studies included in this review have their own limitations. To start with, TORS needs specific training to develop the required skill expertise for the best outcome; however, bias due to the possible impact of the operator’s skills on the surgical outcome were not discussed extensively in any of those studies, except for the study by Arora et al. [19] where it was identified as a possible bias. Other identified limitations included small sample sizes [13, 16, 19–21, 24, 29, 30], selection bias due to inclusion of newly diagnosed patients [19, 20, 23, 24], and absence of control group in some studies [15, 19, 21]. Additionally, the comparison group in most of the included studies underwent adjuvant therapy under various management protocols, and this could have significantly affected the post-operative QOL they reported [17, 18, 22]. Varying survey time points [13, 15, 17, 22, 29], lack of pre-operative QOL data [13, 27, 28] and lack of long-term follow up [21] were also mentioned as possible limitations in the included studies.

Aside from the limitations of the included studies, this scoping review itself also has its own limitations. First, all articles considered for this scoping review were in English; hence, the possibility of non-inclusion of articles published in other languages could not be overruled in this review. Also, the variations in the study characteristics (such as study design) as well as those variations concerning the evaluated QOL aspects in the included studies may have limited the opportunity for a more extensive comparison of the outcomes reported in the included studies. Most of the reviewed assessments were also based on patient-reported outcomes; hence, the possibility of recall bias may exist in such outcomes, making the mapped evidence not absolutely accurate due to this possible bias. Furthermore, this review did not find any relevant randomized controlled trial for inclusion. However, randomized controlled trials are considered the gold standard in evaluating a treatment outcome; hence, the conclusion of this review should be interpreted with caution.

## Conclusion

Within the limitations in this scoping review, compared to the conventional treatment modalities, TORS has demonstrated better quality of life, mostly in the domains related to oral functions such as swallowing and speech, among patients treated with such. This improvement was most evident within the initial post-operative year. However, it is worth noting the heterogeneity of the study designs and the applied QOL instruments in the existing literature. Thus, properly designed prospective longitudinal cohort studies as well as randomized controlled trials, assessing similar aspects in QOL, would be needed to provide better evidence.

### Supplementary Information

**Supplementary Material 1. **

## Data Availability

The datasets generated and/or analyzed during the current study are available from the corresponding author on reasonable request.

## Abbreviations

TORS: Trans-Oral Robotic Surgery
OSA: Obstructive Sleep Apnoea
OPC: Oro-Pharyngeal Carcinoma
CA: Carcinoma
CRT: Chemo-Radiotherapy
RT: Radiotherapy
