# Supplementary material for: Quality of life of patients treated with robotic surgery in the oral and maxillofacial region: a scoping review of empirical evidence
Source: BMC Oral Health. 2024 Feb 26;24:276. doi: 10.1186/s12903-024-04035-w (PMC10895822; doi:10.1186/s12903-024-04035-w)
Supplement: Supplementary file 1 — Supplementary Material 1. [file 12903_2024_4035_MOESM1_ESM.docx]

**SUPPLEMENTARY FILE**

**Table S1. Search string for PubMed database search**

| **Tag** | **Subject Search** | **Search String** |
| --- | --- | --- |
| #1 | Robotic | (Robot[Title/Abstract]) OR (Robotic[Title/Abstract]) |
| #2 | Dental surgery | (((dental surgery[Title/Abstract]) OR (oral surgery[Title/Abstract])) OR (maxillofacial surgery[Title/Abstract])) OR (periodontal surgery[Title/Abstract]) |
| #3 | Quality of life | (Quality of life [Title/Abstract]) OR (wellbeing[Title/Abstract]) |
| #4 | #1 AND #2 AND #3 | (#1) AND (#2) AND (#3) |

**Table S2. Search string for SCOPUS database search**

| **Tag** | **Subject Search** | **Search String** |
| --- | --- | --- |
| #1 | Robotic | (TITLE-ABS-KEY ( robot )  OR  TITLE-ABS-KEY ( robotic ) ) |
| #2 | Dental surgery | ( TITLE-ABS-KEY ( dental  AND surgery )  OR  TITLE-ABS-KEY ( oral  AND surgery )  OR  TITLE-ABS-KEY ( maxillofacial  AND surgery )  OR  TITLE-ABS-KEY ( periodontal  AND surgery ) ) |
| #3 | Quality of life | (TITLE-ABS-KEY ( quality  AND of  AND life )  OR  TITLE-ABS-KEY ( wellbeing ) ) |
| #4 | #1 AND #2 AND #3 | (#1) AND (#2) AND (#3) |

**Table S3. Search string for other database (APA PsycInfo and CINAHL Complete) search via EBSCOHost interface**

| **Tag** | **Subject Search** | **Search String** |
| --- | --- | --- |
| S1 | Robot | AB Robot OR AB Robotic |
| S2 | Dental surgery | AB dental surgery OR AB oral surgery OR AB maxillofacial surgery OR AB periodontal surgery |
| S3 | Quality of life | AB quality of life OR AB wellbeing |
| S4 | S1 AND S2 AND S3 | S1 AND S2 AND S3 |

**Table S4. List of literature subjected to full-text screening and their screening outcomes**

| **No.** | **Citation** | **Outcome** | |
| --- | --- | --- | --- |
|  |  | **Include** | **Exclude (Reasons)** |
| 1 | Barbon CEA, Peterson CB, Moreno AC, Lai SY, Reddy JP, Sahli A, Martino R, Johnson FM, Fuller CD, Hutcheson KA. Adhering to Eat and Exercise Status During Radiotherapy for Oropharyngeal Cancer for Prevention and Mitigation of Radiotherapy-Associated Dysphagia. JAMA Otolaryngol Head Neck Surg. [2022](tel:2022) Oct 1;[148](tel:148)(10):[956-964](tel:956-964). doi: [10.1001](tel:10.1001)/jamaoto.[2022.2313](tel:2022.2313). PMID: [36074459](tel:36074459); PMCID: PMC[9459910](tel:9459910). |  | Yes (Wrong study outcome) |
| 2 | Price K, Van Abel KM, Moore EJ, Patel SH, Hinni ML, Chintakuntlawar AV, Graner D, Neben-Wittich M, Garces YI, Price DL, Janus JR, Foster NR, Ginos BF, Foote RL, Ma D. Long-Term Toxic Effects, Swallow Function, and Quality of Life on MC[1273](tel:1273): A Phase 2 Study of Dose De-escalation for Adjuvant Chemoradiation in Human Papillomavirus-Positive Oropharyngeal Cancer. Int J Radiat Oncol Biol Phys. [2022](tel:2022) Oct 1;[114](tel:114)(2):[256-265](tel:256-265). doi: [10.1016](tel:10.1016)/[j.ijrobp](http://j.ijrobp).[2022.05.047](tel:2022.05.047). Epub [2022](tel:2022) Jun 5. PMID: [35675850](tel:35675850) | Yes |  |
| 3 | Lee E, Gorelik D, Crowder HR, Badger C, Schottler J, Li NW, Siegel R, Sadeghi N, Goodman JF, Thakkar PG, Joshi AS. Swallowing Function Following Neoadjuvant Chemotherapy and Transoral Robotic Surgery for Oropharyngeal Carcinoma: A 2-Year Follow-up. Otolaryngol Head Neck Surg. [2022](tel:2022) Aug;[167](tel:167)(2):[298-304](tel:298-304). doi: [10.1177](tel:10.1177)/[01945998211057430](tel:01945998211057430). Epub [2021](tel:2021) Nov 9. PMID: [34752157](tel:34752157). | Yes |  |
| 4 | Nichols AC, Theurer J, Prisman E, Read N, Berthelet E, Tran E, Fung K, de Almeida JR, Bayley A, Goldstein DP, Hier M, Sultanem K, Richardson K, Mlynarek A, Krishnan S, Le H, Yoo J, MacNeil SD, Winquist E, Hammond JA, Venkatesan V, Kuruvilla S, Warner A, Mitchell S, Chen J, Corsten M, Johnson-Obaseki S, Odell M, Parker C, Wehrli B, Kwan K, Palma DA. Randomized Trial of Radiotherapy Versus Transoral Robotic Surgery for Oropharyngeal Squamous Cell Carcinoma: Long-Term Results of the ORATOR Trial. J Clin Oncol. [2022](tel:2022) Mar 10;40(8):[866-875](tel:866-875). doi: [10.1200](tel:10.1200)/JCO.[21.01961](tel:21.01961). Epub [2022](tel:2022) Jan 7. PMID: [34995124](tel:34995124). |  | Yes (Inaccessible full text) |
| 5 | Fradet L, Charters E, Gao K, Froggatt C, Palme C, Riffat F, Nguyen K, Wu R, Milross C, Clark JR. Avoidance of primary site adjuvant radiotherapy following transoral robotic surgery: a cohort study. ANZ J Surg. [2022](tel:2022) Mar;92(3):[511-517](tel:511-517). doi: [10.1111](tel:10.1111)/ans.[17463](tel:17463). Epub [2022](tel:2022) Jan 11. PMID: [35018703](tel:35018703) |  | Yes (Wrong study outcome) |
| 6 | Salmon KM, Ruiz C, Cognetti DM, Curry JM, Luginbuhl AJ, Bar-Ad V, Leiby BE. Functional Swallow-Related Outcomes Following Transoral Robotic Surgery for Base of Tongue Carcinoma. Dysphagia. [2022](tel:2022) Feb;37(1):[28-36](tel:28-36). doi: [10.1007](tel:10.1007)/s[00455-021-10246](tel:00455-021-10246)-y. Epub [2021](tel:2021) Jan 25. PMID: [33492468](tel:33492468) | Yes |  |
| 7 | Scott SI, Madsen AKØ, Rubek N, Charabi BW, Wessel I, Jensen CV, Friborg J, von Buchwald C. Dysphagia and QoL 3 Years After Treatment of Oropharyngeal Cancer With TORS or Radiotherapy. Laryngoscope. [2022](tel:2022) Oct 14. doi: [10.1002](tel:10.1002)/lary.[30410](tel:30410). Epub ahead of print. PMID: [36239625](tel:36239625) | Yes |  |
| 8 | Achim V, Bolognone RK, Palmer AD, Graville DJ, Light TJ, Li R, Gross N, Andersen PE, Clayburgh D. Long-term Functional and Quality-of-Life Outcomes After Transoral Robotic Surgery in Patients With Oropharyngeal Cancer. JAMA Otolaryngol Head Neck Surg. 2018 Jan 1;144(1):18-27. doi: 10.1001/jamaoto.2017.1790. PMID: 29075740; PMCID: PMC5833591. | Yes |  |
| 9 | Di Luca M, Iannella G, Montevecchi F, Magliulo G, De Vito A, Cocuzza S, Maniaci A, Meccariello G, Cammaroto G, Sgarzani R, Ferlito S, Vicini C. Use of the transoral robotic surgery to treat patients with recurrent lingual tonsillitis. Int J Med Robot. [2020](tel:2020) Aug;16(4):e[2106](tel:2106). doi: [10.1002](tel:10.1002)/rcs.[2106](tel:2106). Epub [2020](tel:2020) Apr 2. PMID: [32223059](tel:32223059). | Yes |  |
| 10 | Xu MJ, Plonowska KA, Gurman ZR, Humphrey AK, Ha PK, Wang SJ, El-Sayed IH, Heaton CM, George JR, Yom SS, Algazi AP, Ryan WR. Treatment modality impact on quality of life for human papillomavirus-associated oropharynx cancer. Laryngoscope. [2020](tel:2020) Feb;[130](tel:130)(2):E48-E56. doi: [10.1002](tel:10.1002)/lary.[27937](tel:27937). Epub [2019](tel:2019) Mar 27. PMID: [30919470](tel:30919470). | Yes |  |
| 11 | Gallitto M, Sindhu K, Wasserman I, De B, Gupta V, Miles BA, Genden EM, Posner M, Misiukiewicz K, Bakst RL. Trimodality therapy for oropharyngeal cancer in the TORS era: Is there a cohort that may benefit? Head Neck. [2019](tel:2019) Sep;41(9):[3009-3022](tel:3009-3022). doi: [10.1002](tel:10.1002)/hed.[25779](tel:25779). Epub [2019](tel:2019) Apr 17. PMID: [30997703](tel:30997703). | Yes |  |
| 12 | Lazarus CL, Ganz C, Ru M, Miles BA, Kotz T, Chai RL. Prospective instrumental evaluation of swallowing, tongue function, and QOL measures following transoral robotic surgery alone without adjuvant therapy. Head Neck. [2019](tel:2019) Feb;41(2):[322-328](tel:322-328). doi: [10.1002](tel:10.1002)/hed.[25455](tel:25455). Epub [2018](tel:2018) Dec 15. PMID: [30552845](tel:30552845). | Yes |  |
| 13 | Benazzo M, Canzi P, Mauramati S, Sovardi F, Occhini A, Maiorano E, Trisolini G, Morbini P. Transoral Robot-Assisted Surgery in Supraglottic and Oropharyngeal Squamous Cell Carcinoma: Laser Versus Monopolar Electrocautery. J Clin Med. [2019](tel:2019) Dec 7;8(12):[2166](tel:2166). doi: [10.3390](tel:10.3390)/jcm[8122166](tel:8122166). PMID: [31817848](tel:31817848); PMCID: PMC[6947577](tel:6947577) |  | Yes (Wrong study outcome) |
| 14 | Ozbay I, Yumusakhuylu AC, Sethia R, Wei L, Old M, Agrawal A, Teknos T, Ozer E. One-year quality of life and functional outcomes of transoral robotic surgery for carcinoma of unknown primary. Head Neck. [2017](tel:2017) Aug;39(8):[1596-1602](tel:1596-1602). doi: [10.1002](tel:10.1002)/hed.[24801](tel:24801). Epub [2017](tel:2017) May 17. PMID: [28513895](tel:28513895). | Yes |  |
| 15 | Fang TJ, Lee LA, Huang BS, Lin CY, Hsu CL, Chang JT, Yen TC, Liao CT, Chiang HC. What should we expect from robotic surgery for second primary oropharyngeal cancer? Eur Arch Otorhinolaryngol. [2017](tel:2017) Aug;[274](tel:274)(8):[3161-3168](tel:3161-3168). doi: [10.1007](tel:10.1007)/s[00405-017-4594-8](tel:00405-017-4594-8). Epub [2017](tel:2017) May 9. PMID: [28484837](tel:28484837). |  | Yes (Wrong study outcome) |
| 16 | Rodin D, Caulley L, Burger E, Kim J, Johnson-Obaseki S, Palma D, Louie AV, Hansen A, O'Sullivan B. Cost-Effectiveness Analysis of Radiation Therapy Versus Transoral Robotic Surgery for Oropharyngeal Squamous Cell Carcinoma. Int J Radiat Oncol Biol Phys. [2017](tel:2017) Mar 15;97(4):[709-717](tel:709-717). doi: [10.1016](tel:10.1016)/[j.ijrobp](http://j.ijrobp).[2016.11.029](tel:2016.11.029). Epub [2016](tel:2016) Nov 27. PMID: [28244405](tel:28244405). |  | Yes (Wrong study outcome) |
| 17 | Ling DC, Chapman BV, Kim J, Choby GW, Kabolizadeh P, Clump DA, Ferris RL, Kim S, Beriwal S, Heron DE, Duvvuri U. Oncologic outcomes and patient-reported quality of life in patients with oropharyngeal squamous cell carcinoma treated with definitive transoral robotic surgery versus definitive chemoradiation. Oral Oncol. [2016](tel:2016) Oct;61:[41-6](tel:41-6). doi: [10.1016](tel:10.1016)/[j.oraloncology](http://j.oraloncology).[2016.08.004](tel:2016.08.004). Epub [2016](tel:2016) Aug 25. PMID: [27688103](tel:27688103); PMCID: PMC[7717075](tel:7717075). | Yes |  |
| 18 | Arora A, Chaidas K, Garas G, Amlani A, Darzi A, Kotecha B, Tolley NS. Outcome of TORS to tongue base and epiglottis in patients with OSA intolerant of conventional treatment. Sleep Breath. [2016](tel:2016) May;20(2):[739-47](tel:739-47). doi: [10.1007](tel:10.1007)/s[11325-015-1293-9](tel:11325-015-1293-9). Epub [2015](tel:2015) Dec 15. PMID: [26669877](tel:26669877). | Yes |  |
| 19 | Mercante G, Masiello A, Sperduti I, Cristalli G, Pellini R, Spriano G. Quality of life and functional evaluation in patients with tongue base tumors treated exclusively with transoral robotic surgery: A 1-year follow-up study. J Craniomaxillofac Surg. [2015](tel:2015) Oct;43(8):[1561-6](tel:1561-6). doi: [10.1016](tel:10.1016)/[j.jcms](http://j.jcms).[2015.06.024](tel:2015.06.024). Epub [2015](tel:2015) Jun 27. PMID: [26197796](tel:26197796). | Yes |  |
| 20 | Chen AM, Daly ME, Luu Q, Donald PJ, Farwell DG. Comparison of functional outcomes and quality of life between transoral surgery and definitive chemoradiotherapy for oropharyngeal cancer. Head Neck. [2015](tel:2015) Mar;37(3):[381-5](tel:381-5). doi: [10.1002](tel:10.1002)/hed.[23610](tel:23610). Epub [2014](tel:2014) Apr 3. PMID: [24431059](tel:24431059). | Yes |  |
| 21 | Hans S, Hoffman C, Croidieu R, Vialatte de Pemille G, Crevier-Buchman L, Monfrais-Pfauwadel MC, Menard M, Brasnu D. Evaluation of quality of life and swallowing in patients with cancer of the oropharynx treated with assisted transoral robotic surgery. Rev Laryngol Otol Rhinol (Bord). [2013](tel:2013);[134](tel:134)(1):[49-56](tel:49-56). PMID: [24494332](tel:24494332). |  | Yes (Wrong language) |
| 22 | Park YM, Kim WS, Byeon HK, De Virgilio A, Lee SY, Kim SH. Clinical outcomes of transoral robotic surgery for head and neck tumors. Ann Otol Rhinol Laryngol. [2013](tel:2013) Feb;[122](tel:122)(2):[73-84](tel:73-84). doi: [10.1177](tel:10.1177)/[000348941312200202](tel:000348941312200202). PMID: [23534121](tel:23534121). |  | Yes (Wrong study outcome) |
| 23 | Leonhardt FD, Quon H, Abrahão M, O'Malley BW Jr, Weinstein GS. Transoral robotic surgery for oropharyngeal carcinoma and its impact on patient-reported quality of life and function. Head Neck. [2012](tel:2012) Feb;34(2):[146-54](tel:146-54). doi: [10.1002](tel:10.1002)/hed.[21688](tel:21688). Epub [2011](tel:2011) Apr 5. PMID: [21469248](tel:21469248). | Yes |  |
| 24 | Sinclair CF, McColloch NL, Carroll WR, Rosenthal EL, Desmond RA, Magnuson JS. Patient-perceived and objective functional outcomes following transoral robotic surgery for early oropharyngeal carcinoma. Arch Otolaryngol Head Neck Surg. [2011](tel:2011) Nov;[137](tel:137)(11):[1112-6](tel:1112-6). doi: [10.1001](tel:10.1001)/archoto.[2011.172](tel:2011.172). PMID: [22106235](tel:22106235) | Yes |  |
| 25 | Genden EM, Kotz T, Tong CC, Smith C, Sikora AG, Teng MS, Packer SH, Lawson WL, Kao J. Transoral robotic resection and reconstruction for head and neck cancer. Laryngoscope. [2011](tel:2011) Aug;[121](tel:121)(8):[1668-74](tel:1668-74). doi: [10.1002](tel:10.1002)/lary.[21845](tel:21845). PMID: [21792953](tel:21792953). | Yes |  |
| 26 | Vicini C, Dallan I, Canzi P, Frassineti S, La Pietra MG, Montevecchi F. Transoral robotic tongue base resection in obstructive sleep apnoea-hypopnoea syndrome: a preliminary report. ORL J Otorhinolaryngol Relat Spec. [2010](tel:2010);72(1):[22-7](tel:22-7). doi: [10.1159](tel:10.1159)/[000284352](tel:000284352). Epub [2010](tel:2010) Feb 18. PMID: [20173358](tel:20173358). | Yes |  |
| 27 | Meccariello G, Montevecchi F, D'Agostino G, Iannella G, Calpona S, Parisi E, Costantini M, Cammaroto G, Gobbi R, Firinu E, Sgarzani R, Nestola D, Bellini C, De Vito A, Amadori E, Vicini C. Trans-oral robotic surgery for the management of oropharyngeal carcinomas: a 9-year institutional experience. Acta Otorhinolaryngol Ital. [2019](tel:2019) Apr;39(2):[75-83](tel:75-83). doi: [10.14639](tel:10.14639)/[0392-100](tel:0392-100)X-[2199](tel:2199). PMID: [31097824](tel:31097824); PMCID: PMC[6522856](tel:6522856). |  | Yes (Wrong study outcome) |
